# The VISA-C Questionnaire: A Self-Administered Assessment to Measure Finger/Hand/Wrist Pain in Climbers

**DOI:** 10.1186/s40798-025-00912-y

**Published:** 2025-10-01

**Authors:** Natalie K. Gilmore, Peter Klimek, Emil Abrahamsson, Keith Baar

**Affiliations:** 1 Department of Neurobiology, Physiology and Behavior, University of California Davis, One Shields Ave, 195 Briggs Hall, Davis, CA 95616 USA; 2 Crimpd, Inc., Seattle, WA 98118 USA; 3 Stories and Stones, Ängarna, 837 98 Sweden; 4 Department of Physiology and Membrane Biology, University of California Davis, Davis, CA 95616 USA

**Keywords:** Rock climbing, Exercise, Tendon, Finger strength, Injury risk, Pain

## Abstract

**Background:**

Rock climbing places high loads through the hands, wrists, and fingers, leading to high injury rates, with the highest proportion in the fingers. Until now, there has been no attempt to categorize pain in the forearm to assess readiness to train. The purpose of this study was to create a questionnaire, the VISA-C (Victorian Institute of Sports-like Assessment; C for climbing), to measure forearm pain and determine how pain limits training.

**Methods:**

We asked rock climbers aged 18 to 50 years old, who met the participation criteria and gave consent, to complete a survey containing 8 questions. We analyzed data from climbers who responded to the online questionnaire in the 9-week period between November 5, 2024, and January 8, 2025. We obtained a diverse international sample with English-speaking respondents from 54 countries. We included a supplementary questionnaire to compare the survey results against participant demographics, lifestyle, health, and sport-specific history and habits.

**Results:**

We analyzed data from 1,110 climbers who completed the form. VISA-C scores were significantly different as a function of pain. The mean VISA-C score of the group with no pain was the highest (83.21/100), lower in the group reporting some pain (72.28/100), and lowest in the most severe group with activity-limiting pain (60.05/100), indicating our questionnaire scales with pain severity. We then used the secondary data gathered on our participants to search for associations between pain or skill level and demographic, health, and training habits. Of these, only blood pressure was associated with differences in VISA-C scores.

**Conclusions:**

VISA-C score scaled with pain and can be compared broadly across all major demographics. We observed interesting trends in our secondary analyses. Several variables correlated significantly with either VISA-C score or climbing skill level, but none correlated well with both. Many of the variables we compared agreed with existing literature or pointed to novel associations that warrant more investigation.

**Supplementary Information:**

The online version contains supplementary material available at 10.1186/s40798-025-00912-y.

## Key Points

• We determined whether an 8-question survey, the VISA-C, could measure finger, hand, and wrist pain and the associated limitations on performance in rock climbers.

• VISA-C scores were not different between all major demographic groups, indicating the questionnaire can be used broadly.

• VISA-C score scaled with blood pressure: those with elevated blood pressure had a higher VISA-C score (fewer injuries).

• Climbing skill level was higher in men, boulderers, athletes who climbed > 5 years or > 10 h per week, and for those who climb outside for some of their training.

• No demographic measure was associated with both a change in VISA-C and skill.

**Supplementary Information:**

The online version contains supplementary material available at 10.1186/s40798-025-00912-y.

## Introduction

Rock climbing is an increasingly popular sport worldwide that places uniquely high loads on the finger flexor system and musculoskeletal tissues of the hand and wrists. Movements in the sport are complex, and performance requires the coordination of different physiological skills with the critical involvement of many different tissues. Climbers are a unique population to study connective tissue injury, recovery, and physiological adaptations to training. There are several physiological characteristics linked with skilled and elite climbers. Climbing is a strength-to-weight ratio sport. Strength of the forearm muscles relative to body mass, endurance of the forearm muscles, and climbing-specific flexibility are all factors likely to contribute to climbing skill level [1]. Climbing injuries are common: one retrospective study found that half of the participants in their study sustained an injury in the past year [2]. Common modes of injury were falls (10%), chronic overuse (33%) and acute injuries caused by strenuous climbing moves (28%). Fingers are the most commonly injured body part in climbers [2–4]. A prospective study examining overuse and acute injuries of approximately 600 climbers lists the incidence of injuries they encountered in the clinic, with pulley ruptures and strains being the most common. However, the ability to perform large-scale (global) clinical studies looking at interventions that decrease injury rate is not currently possible.

To address this clinical gap, the primary purpose of this study was to create and test a self-administered hand and wrist pain questionnaire for rock climbers, which we have called the VISA-C (Victorian Institute of Sports-like Assessment; C for climbing). To date, no standard self-administered questionnaire exists to evaluate the severity of climbing-related connective tissue injury of the hand and wrist region and the readiness to train. This 8-question assessment was designed by modifying the validated and widely used VISA-P (patellar tendon) [5] and VISA-A (Achilles tendon) [6] questionnaires, for evaluating the severity of pain from wrist and finger tendinopathy and pulley injuries and how the level of pain correlates with limits to the intensity and volume of climbing. We kept the general format but modified some of the questions on the VISA-C questionnaire to increase their relevance to climbing, and typical sport-specific training frequency and intensity. Our hypothesis was that this questionnaire would be able to differentiate between climbers: those who are healthy and had no pain would have the highest VISA-C scores, climbers with sport-specific activity-limiting pain would have the lowest, and climbers with some pain but without limits on activity duration or intensity would have an intermediate mean score on the questionnaire. After establishing the VISA-C correlated well with pain, we used secondary data gathered on our participants to search for associations between pain incidence and demographic, health, and lifestyle factors, as well as sport-specific history and habits.

## Methods

### Recruitment Methods

All participants gave written consent to participate in the study protocol that had been approved by the Institutional Review Board at the University of California Davis (protocol #2242178 approved 11/04/2024) and was written in accordance with standards set by the *Declaration of Helsinki*. The questionnaire was administered as a poll using Google forms, meaning that anyone in the world could participate, though the questionnaire was only available in English. We recruited participants by advertising the study online and by sharing the link with known climbing contacts, and as part of a video on Emil Abrahamsson’s YouTube channel. We included people who responded to the poll in the period between November 5, 2024, and January 8, 2025. Over these nine weeks, 1,110 people completed the form out of the 1,188 people who began the survey. Of the 78 individuals excluded, most did not meet the selection criteria, or they did not fully fill out the questions on the survey.

### Selection Criteria

Recruitment targeted rock climbers between 18 and 50 years of age unless they had one or more of the listed exclusion criteria. To participate in the survey, the individual was required to either be (A) healthy and currently climbing an average of at least once a week; or (B) currently have an injury to the hand/wrist region that may limit their climbing intensity or training volume. Climbers with any of the following were excluded: (1) a non-climbing related injury that limited climbing intensity or training volume; (2) injuries in parts of the body other than the forearm (finger/hand/wrist) region that limited climbing intensity or training volume; (3) medical conditions or medications that limited climbing intensity or training volume; or (4) individuals currently climbing/training less than once per week on average for a reason other than a climbing-related injury to the forearm region.

### VISA-C Questionnaire Testing

The format of the original VISA questionnaires VISA-P (patellar tendon) [5] and VISA-A (Achilles tendon) [6] used an unequally weighted 8-question survey, with 10 points each allocated to Questions 1 through 7, and a multiplier placed on Question 8, based on the pain group of each respondent. The questions are answered by selecting a discrete number between 0 and 10, with lower scores on the questions indicating more pain and inability to complete activity. To group our survey respondents for VISA-C testing purposes, we asked respondents to select one of three groups based on their current pain levels, using verbiage similar to that of the other VISA questionnaires: the first group (“No Pain”) had no pain while undertaking finger, hand, and wrist loading; the second (“Pain”) stated that they experience pain, but it does not stop from climbing; and the third group (“Limiting Pain”) had pain that stopped them from climbing or placing load on their fingers, hands, or wrists. Question 8 in the original VISA-C surveys used a 1x multiplier for the Limiting Pain group, a 2x multiplier for the Pain group, and a 3x multiplier for the No Pain group, creating different maximum total point values for each group to presumably create a larger spread of the data between pain groups and reduce the maximum point total for the Pain and Limiting Pain groups.

**Fig. 1:**
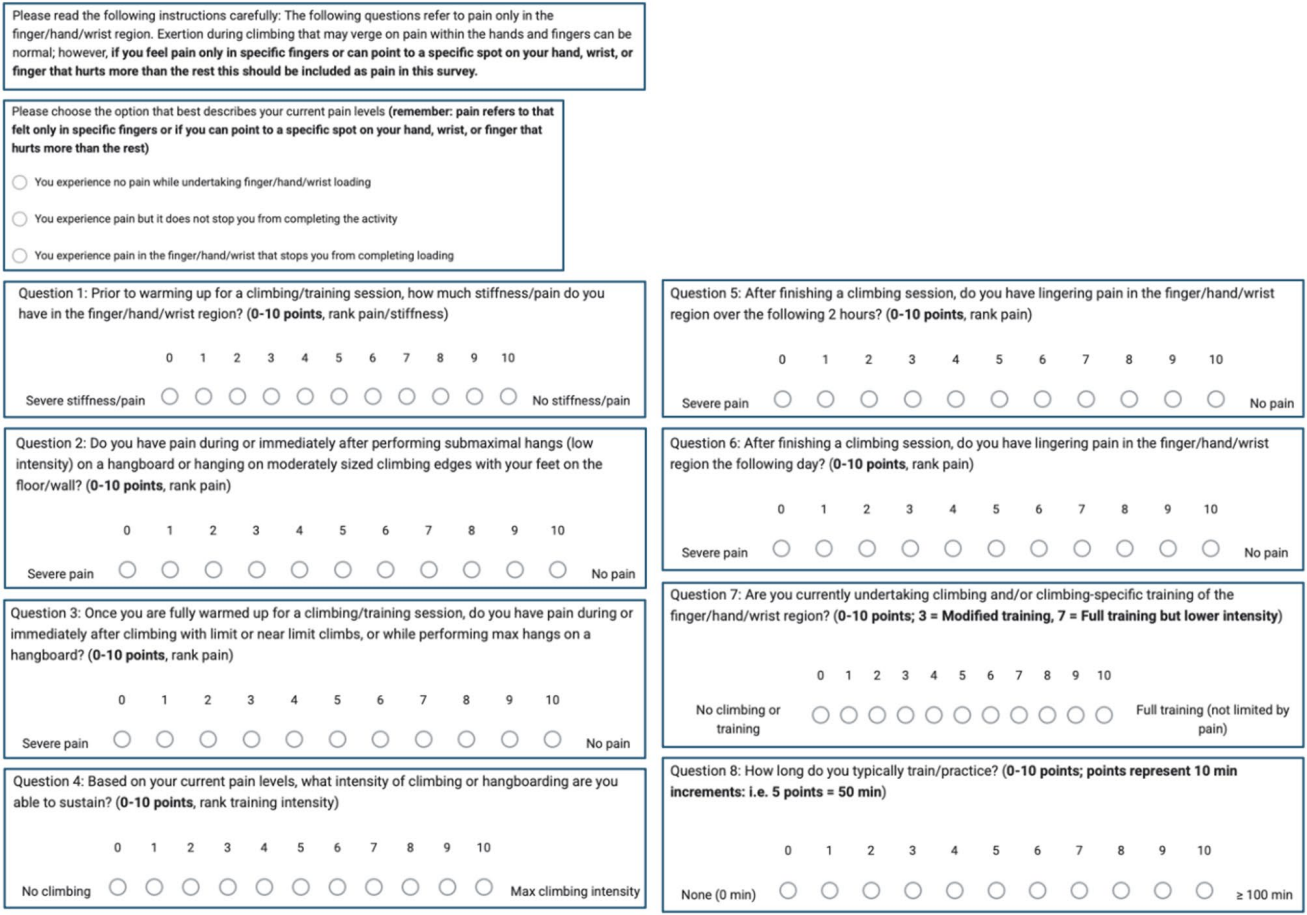
The VISA-C Questionnaire. The instructions and questions for the complete VISA-C questionnaire. Note that the questionnaire is very short (< 5 min to complete) and as such will be extremely useful for research into climbing related forearm injury

The full VISA-C survey is provided in Fig. 1. We adapted the questions to increase relevance to climbing, and we provided the following instructions prior to the survey: “The following questions refer to pain only in the finger/hand/wrist region. Exertion during climbing that may verge on pain within the hands and fingers can be normal; however, if you feel pain only in specific fingers or can point to a specific spot on your hand, wrist, or finger that hurts more than the rest this should be included as pain in this survey.” The specific cue to focus on discrete and localizable pain was because in early testing respondents would all report moderate to high levels of “pain” with maximal loading. Survey respondents were sorted into three pain groups based on their current pain levels. Deviating slightly from the original questionnaires, the VISA-C survey has 8 questions, with discrete responses from 1 to 10, but without the multiplier on Question 8. Each question was worth a maximum of 10 points, for a maximum total possible score of 80 points; we then converted each score into a percentage by dividing by 80 and multiplying by 100 to improve clarity and data visualization. Question 8 in our survey, as in the originals, is targeted to measure the activity duration an athlete can sustain. Specifically, it asks how long a person typically trains/practices for in a single session, with 0–10 for each increasing point representing 10-minute increments with a maximal time of ≥ 100 min. We do not believe this was an appropriate question to weight more heavily than the others in our survey: when we did apply a multiplier, the questionnaire produced a bimodal distribution of scores in all three pain groups. By contrast, equally weighting each of the 8 questions on the VISA-C produced a normal distribution of scores.

### Secondary Questionnaire and Outcomes

In our study questionnaire, we asked participants various supplemental questions and searched for associations to VISA-C score and to climbing skill level. We asked participants to characterize their project grade on the IRCRA scale to quantify their skill level. We asked about climbing habits, including the number of years they have participated in the sport, the number of hours they typically train per week (past 6 months), their preferred climbing discipline (bouldering, sport, or both) and where they typically climb (gym only, mostly gym, equal gym/outdoors, or mostly outdoor). We also asked whether they had participated in submaximal (i.e. no hangs or “Abrahangs”) and/or maximal (i.e. max hangs) supplemental finger training in the past six months. Participant demographics we asked participants for were biological sex, age, race, height, weight, their typical diet (meat eater, vegetarian, or vegan diet) and blood pressure (asking participants to identify their blood pressure classification using a scale from the American Heart Association (normal = < 120, elevated = 120–129, high blood pressure = 130 or above). BMI was obtained based on participant-reported height and weight in either imperial or metric scales and converted to metric, then calculated using the formula (BMI = weight (kg) / height (cm) [2]).

### Data Analysis

**Fig. 2:**
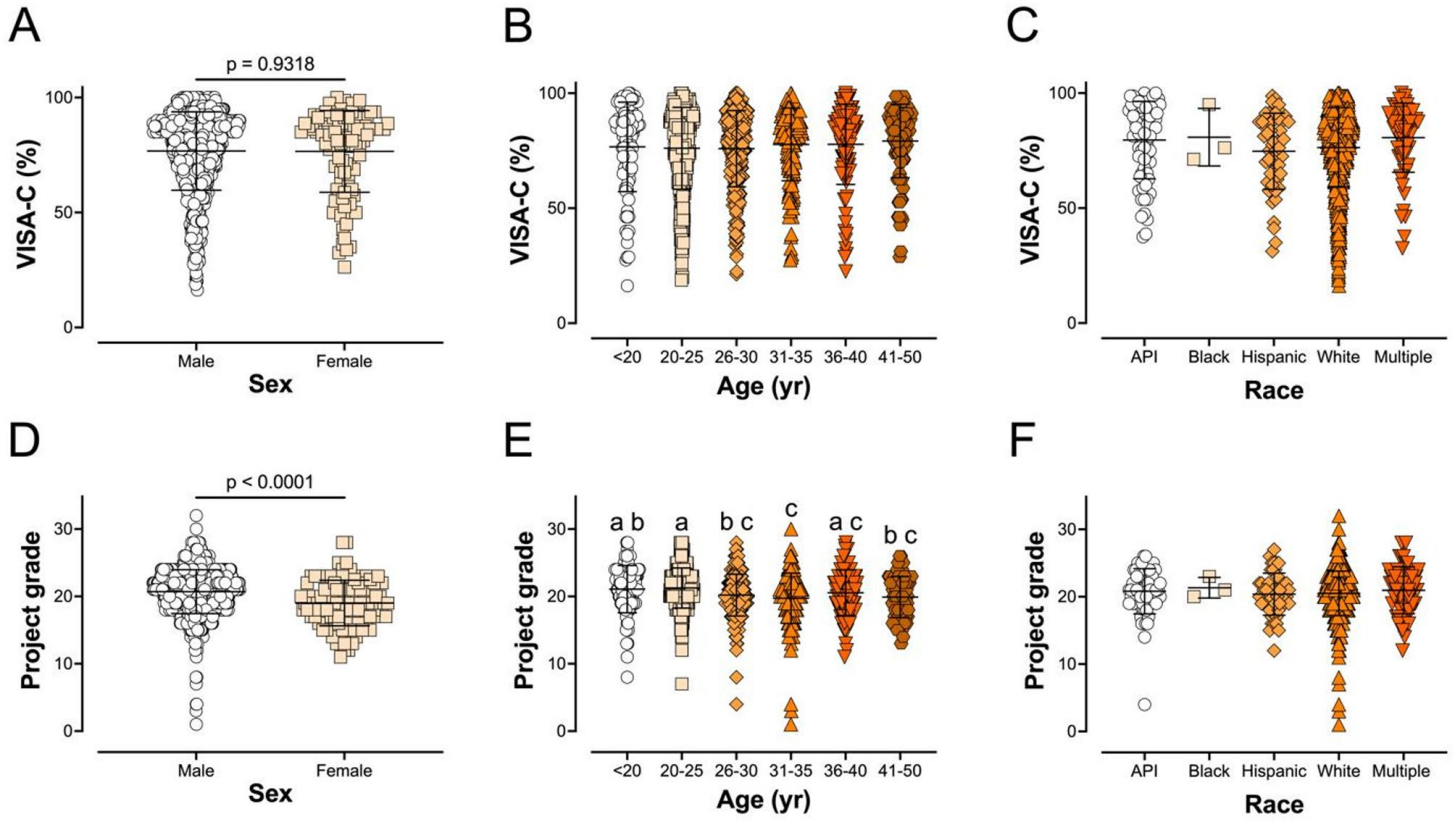
Impact of respondent demographics on VISA-C pain score and skill level (IRCRA project grade). Sex (**A**, **D**), age (in years; **B**, **E**), and race (**C**, **F**) were compared. (**A**) VISA-C scores of males and females were not statistically different (unpaired t-test *p* = 0.9318). (**B**) VISA-C score was not statistically different for age (correlation *p* = 0.1196; one-way ANOVA for the age ranges listed in the figure *p* = 0.5230). (**C**) VISA-C scores were not different for the racial groups: Asian/Pacific Islander (API), Black, Hispanic, White/Caucasian, and Multiple/other (one-way ANOVA *p* = 0.1654). (**D**) Males had higher project grades (mean = 20.7) than females (mean = 19.0) (unpaired t-test: *p* < 0.0001). (**E**) Project grade decreased with age (correlation *p* < 0.0001; one-way ANOVA for the age ranges listed in the figure *p* < 0.0001). (**F**) Race was not a statistically significant predictor of project grade (one-way ANOVA *p* = 0.7624)

Anyone who met the selection criteria and gave written consent was included in our analysis. People who only partially completed the essential information were omitted from analysis: those who did not completely answer the VISA-C were omitted entirely, and those who omitted auxiliary questions or gave improbable or impossible answers due to question comprehension or input errors (i.e., hours per week was more than the total number of hours in a week) were omitted from analysis of those individual parameters. Because VISA-C scores were not different by most demographic measures including biological sex (Fig. 2A), age (Fig. 2B) or project grade (Fig. S1A), for analysis we pooled participants of both sexes, all ages (18–50 year) and all climbing ability levels.

### Statistics

Statistical analysis was performed using GraphPad Prism, version 10.1 (San Diego, CA, USA). Tables 1, 2 and 3 report group numbers (n) and mean VISA-C and project grades for all categories. Statistical analyses and significance levels are described in the figures or figure legends. All individual data points are shown on the graphs. A *p*-value of < 0.05 was set a priori for statistical significance. Correlations were performed when comparing two numerical variables, with results listed in Table 4. Ordinary one-way ANOVA was performed when comparing multiple groups against VISA-C or against IRCRA project grade; ANOVA results are listed in Table 5. Multiple comparisons were made using Tukey’s *post hoc* test. Compact letter display (CLD) was used to display pairwise comparisons from one-way ANOVAs on figures: all groups were assigned one or more letter variables, sorted by group mean, with the highest mean assigned the letter “a”. If two groups share a letter, it indicates that the *p*-value of the pairwise comparison was not significant. Groups with different letters were significantly different from one another.

**Table 1:** Respondent categorization of pain and demographic groups

Category	Group/Bin	*n*	VISA-C	Project grade
Pain group	No pain	542	83.2	20.5
	Pain	491	72.3	20.6
	Limiting Pain	77	60.1	20.6
Sex	Male	1,003	76.7	20.7
	Female	107	76.6	19.0
Age (yr)	< 20	92	76.7	21.1
	20–25	349	76.1	21.3
	26–30	297	75.9	20.2
	31–35	182	77.8	19.7
	36–40	99	77.8	20.5
	41–50	91	79.2	19.9
Race	API	68	79.6	20.8
	Black	3	80.8	20.3
	Hispanic	50	74.7	20.4
	White	922	76.4	20.5
	Multiple/Other	65	80.6	21.0
Nationality	Africa	3		
	Asia	34		
	Europe	687		
	North America	329		
	Oceania	40		
	South America	16		

A total of 1,110 participants took part in the study. Means for VISA-C score (%) and IRCRA project grade are reported. N = number of participants per group

**Table 2:** Categorization of measures of health and lifestyle

Category	Group/Bin	*n*	VISA-C	Project grade
BMI	≤ 19	60	77.9	20.3
	19–21	197	76.5	20.7
	21–23	409	78.1	21.1
	23–25	271	76.9	20.4
	25–27	118	77.5	19.6
	> 27	52	72.3	18.5
Diet	Meat	892	76.3	20.6
	Vegetarian	167	78.0	20.1
	Vegan	49	81.5	20.6
BP	Normal	972	76.4	20.5
	Elevated	103	82.5	20.4
	High	12	83.1	22.0

BMI = body mass index, bp = blood pressure

**Table 3:** Categorization of participant climbing history and habits

Category	Group/Bin	*n*	VISA-C	Project grade
Years climbing	≤ 1	138	75.1	18.6
	1–5	470	76.0	20.1
	5–10	306	77.2	21.4
	10–15	100	77.7	21.2
	15–20	39	82.4	22.5
	> 20	55	80.6	22.1
Hours climb	< 5	253	77.2	19.3
per week	5–10	635	76.4	20.5
	10–15	156	77.5	21.9
	15–20	35	80.9	22.9
	≥ 20	20	73.5	22.9
Discipline	Boulder	754	76.0	20.9
	Sport/Toprope	114	78.5	18.7
	Both	241	78.2	20.2
Location	Only gym	280	75.0	19.5
	Mostly gym	585	77.1	20.5
	Equal	191	77.5	21.8
	Mostly outdoor	53	79.1	21.6

**Table 4:** Correlations. *P*-values are reported

Category	vs. VISA-C	vs. project grade
Project grade	0.0570	---
Age	0.1196	**< 0.0001**
BMI	0.7181	**0.0001**
Years climbing	**0.0045**	**< 0.0001**
Hours climb/wk	0.6067	0.7624

**Table 5:** Group comparisons

Category	vs. VISA-C	vs. project grade
Pain group	**< 0.0001**	0.8784
Sex	0.9318	**< 0.0001**
Age (bins)	0.5230	**< 0.0001**
Race	0.1654	0.7624
BMI (bins)	0.1654	**< 0.0001**
Diet	0.0686	0.7283
BP	**0.0012**	0.2894
Years climbing (bins)	0.0851	**< 0.0001**
Hours climb/wk	0.4870	**< 0.0001**
Discipline	0.1239	**< 0.0001**
Location	0.2129	**< 0.0001**
Project grade (bins)	0.3388	**---**
Recent finger training (all)	0.8292	**---**
Recent finger training (≤ 1 year)	0.6510	**---**

*P*-values are reported. All represent one-way ANOVA except for sex, which was analyzed by unpaired t-test. Bins indicate the data was analyzed using the groupings as listed in Tables 1–3. BMI = body mass index, bp = blood pressure

## Results

### VISA-C Questionnaire Testing

**Fig. 3:**
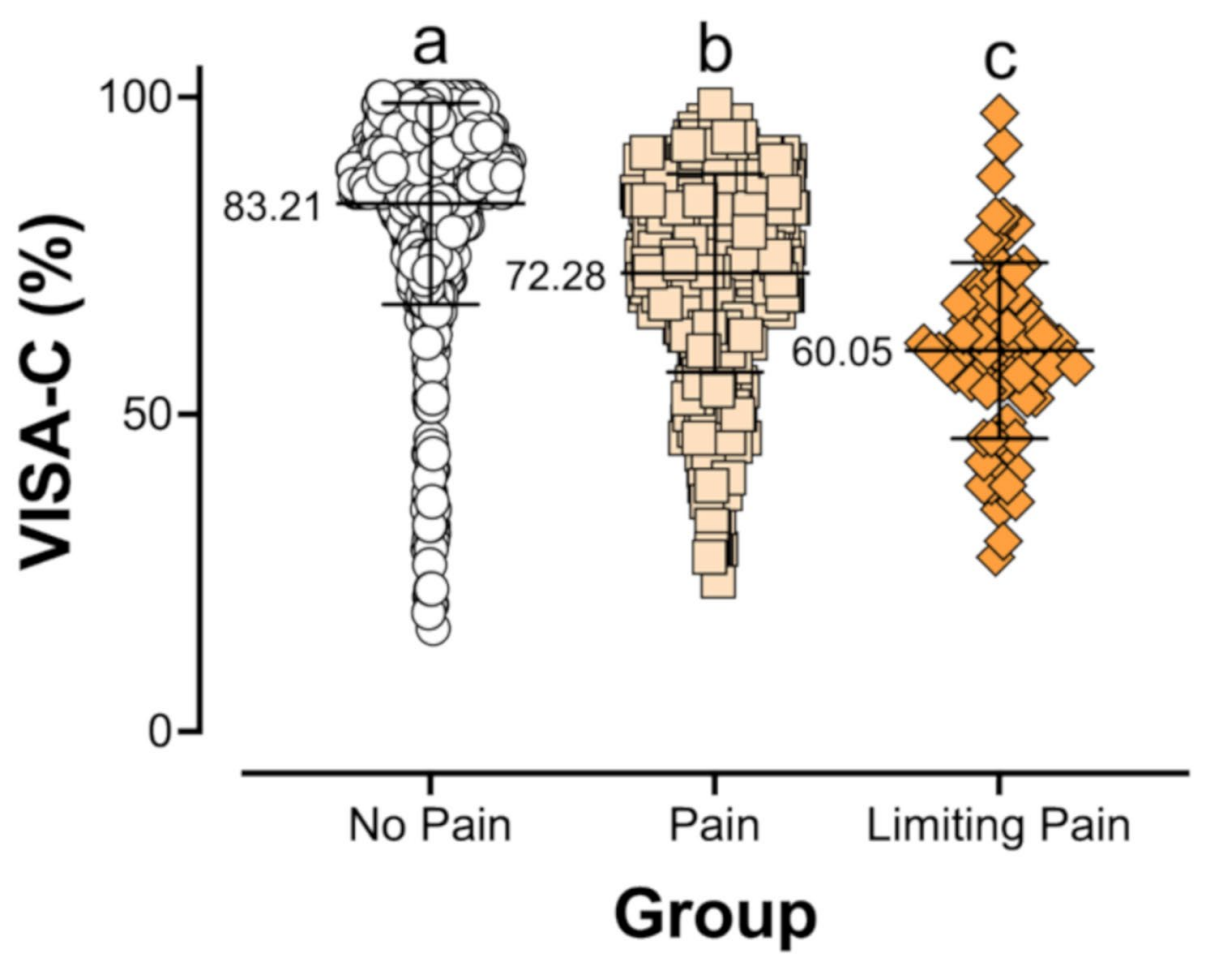
VISA-C scores of respondents categorized into three different self-described pain categories: either “No Pain”, pain that does not stop them from loading (“Pain”), or activity-limiting pain (“Limiting Pain), reflecting the current reported pain levels for each respondent. VISA-C score was highest in the No Pain group (mean = 83.21%), lower in the Pain group (72.28%) and lowest in the Limiting Pain group (60.05%). The group scores were statistically significant (one-way ANOVA, *p* < 0.0001; Tukey’s multiple comparisons: all *p* < 0.0001). Pairwise differences between groups are demarcated in the figure by compact letter display (CLD). Groups with the same letter are not significantly different, while groups with different letters are statistically different from each other

The VISA-C total score was based on the additive score of eight equally weighted pain- and activity-specific questions meant to gauge both how much pain and what training intensity and volume a participant was currently capable of undertaking. The first group (“No Pain”; *n* = 542) stated that they “experience no pain while undertaking finger/hand/wrist loading”. The second (“Pain”; *n* = 491) said that they “experience pain but it does not stop [them] from completing the activity”, and the third group (“Limiting Pain”; *n* = 77) experiencing “pain in the finger/hand/wrist region that stops [them] from completing loading” (Table 1). The results were consistent with our hypothesis and indicate that the VISA-C score is sensitive to pain levels and their limits on sport-specific activity, with mean VISA-C scores for each group (Fig. 3) of 83.2% (No Pain; SD = 15.9), 72.3% (Pain; SD = 15.7) and 60.1% (Limiting Pain; SD = 13.9). All three groups were statistically different from each other (*p* < 0.0001). The highest score recorded was 100% (*n* = 14) and the lowest score was 16.3% (*n* = 1).

To determine whether there were correlations between climbing-related pain and demographics, or other health, and sport-specific variables, we asked participants to answer a supplemental set of questions in conjunction with the VISA-C pain and activity questionnaire. We then ran simple correlations between demographic variables against both VISA-C score and obtained r^2^ values to determine the strength of the correlation. To be clear, the VISA-C score is only a snapshot of the current pain levels for each respondent and not a comprehensive injury history. However, with the large sample of respondents in our study (*n* = 1,110), we can begin to make inferences about climbing-related injury rates at a population level based on average current VISA-C pain scores for each grouping. In addition to comparing these variables against VISA-C score, we also compared them against skill level. For this, the survey had participants use the International Rock Climbing Research Association (IRCRA) rating system, with conversions to all the major grading systems provided, to self-report their skill level (project grade). Our participants spanned the entire ability scale, from Lower Grade (Level 1) to Higher Elite (Level 5), with the highest score reported by any respondents being IRCRA project grade of 30 (corresponding to V14 on the Vermin scale, 8B + on the Font scale, or 9a + on the French/sport scale) out of a maximum of 32.

There was a trend toward a positive correlation between project grade and VISA-C score (*p =* 0.0570; Table 4) but when climbers were binned, there was no difference between any of the groups (*p =* 0.3388; Figure S1A). We also compared recent finger training history after grouping participants based on whether they had performed high/maximal, submaximal/low intensity, or both forms of supplemental finger training in the past six months. There were no significant differences among the VISA-C scores of each recent training history group when we compared either all respondents (*p* = 0.8292; Fig. S1B) or climbers with ≤ 1 year of climbing experience (*p* = 0.6510; Fig. S1C).

### Respondent Demographics

The majority (90%; *n* = 1,003) of participants were male; only 10% were female (*n* = 107). Interestingly, there was no sex difference for VISA-C scores in males (76.7%) vs. females (76.6%) (*p* = 0.9318; Fig. 2A). There was no correlation between age and VISA-C score (*p* = 0.1196; Fig. 2B). There was no statistical difference as a function of racial group, either: Asian/Pacific Islander (79.6%), Black (80.8%), Hispanic (74.7%), White/Caucasian (76.4%), or Multiple/Other (80.6%) (*p* = 0.1654; Fig. 2C).

Sex was a significant predictor of project grade: males had a mean IRCRA level of 20.7 vs. 19.0 for females (*p* < 0.0001; Fig. 2D). For the age range of participants in our study (18–50 years old), age was negatively correlated with skill level (*p* < 0.0001). The specific age ranges with significant differences are shown in Fig. 2E. Project grade was not different as a function of race (*p* = 0.7624; Fig. 2F).

### Health and Lifestyle

**Fig. 4:**
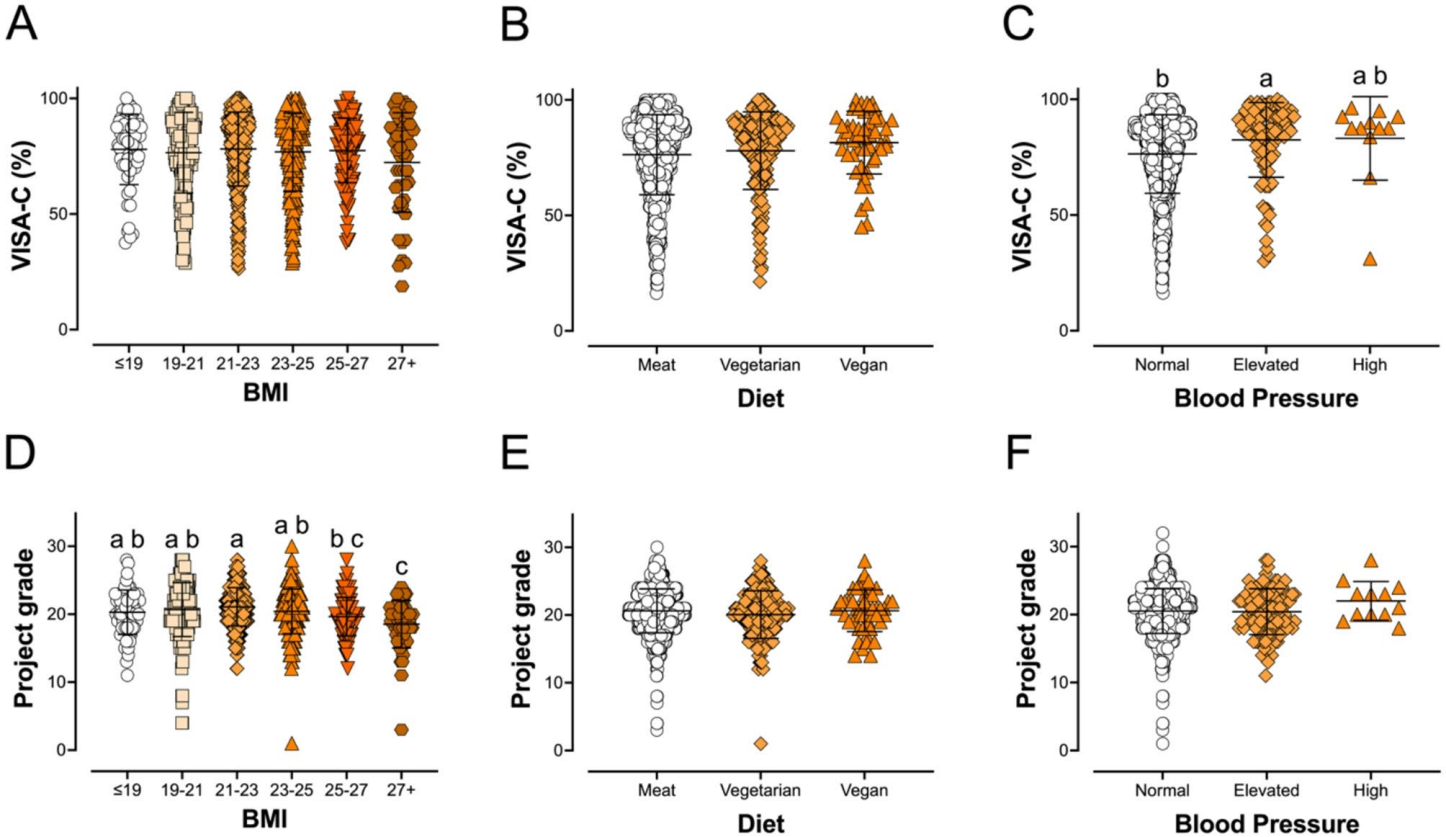
Health and lifestyle factors as predictors of VISA-C score and climbing skill level. Comparing body mass index (BMI; **A**, **D**), diet (**B**, **E**), and blood pressure (BP; **C**, **F**). (**A**) BMI overall was not a predictor of VISA-C score (correlation *p* = 0.7181; one-way ANOVA with the groups in the figure *p* = 0.2654). (**B**) There was a trend toward significance with VISA-C score vs. diet (one-way ANOVA *p* = 0.0686). (**C**) BP was a significant predictor of VISA-C score (one-way ANOVA *p* = 0.0012) with normal BP individuals (mean VISA-C = 76.39%) having lower scores than elevated BP individuals (mean = 82.46%). (**D**) BMI had a negative correlation with project grade (correlation *p* = 0.0001; one-way ANOVA with the groups in the figure *p* < 0.0001). (**E**) Project grade was not different between the three diets (one-way ANOVA *p* = 0.7283). (**F**) Project grade was not statistically different with BP groups (one-way ANOVA *p* = 0.2894)

Next, we looked for trends in pain levels, as well as climbing skill level, as a function of health and lifestyle factors. Body mass index (BMI) was calculated based on participant height and weight (BMI = weight (kg) / height (cm) [2]). Following the standard ranges, our mean BMI was 22.7. 3.3% of our participants were underweight (BMI ≤ 18.5, *n* = 36), 81.4% were normal (BMI 18.5–25; *n* = 901), 14.6% were overweight (BMI = 25–30; *n* = 162), and 0.7% were obese (BMI = ≥ 30; *n* = 8). Participant BMI did not affect VISA-C score (Fig. 2C; *p =* 0.1654). Our climbers’ diets were investigated as well: 81% of participants reporting they eat meat, 15% were vegetarian, and 4% vegan. The mean VISA-C score for meat eaters was 76.3%, 78.0% for vegetarians, and 81.5% for vegans, with a trend toward higher scores for vegetarians and vegans that did not reach significance (*p =* 0.0686; Fig. 4B). Blood pressure (BP) did have a significant effect on VISA-C score (*p* = 0.0012; Fig. 4C). Interestingly, VISA-C scores were higher in those who had elevated vs. normal BP; there were too few high BP individuals in our study (*n* = 12) to statistically analyze for differences with that group.

Even though no relationship was observed between BMI and pain, climbing skill did decrease in the overweight and obese groups vs. low and normal BMIs (Fig. 4D). Neither diet (*p* = 0.7283; Fig. 4E) nor BP (*p* = 0.2894; Fig. 2F) were associated with differences in project grade in our respondent pool.

### Climbing History and Habits

**Fig. 5:**
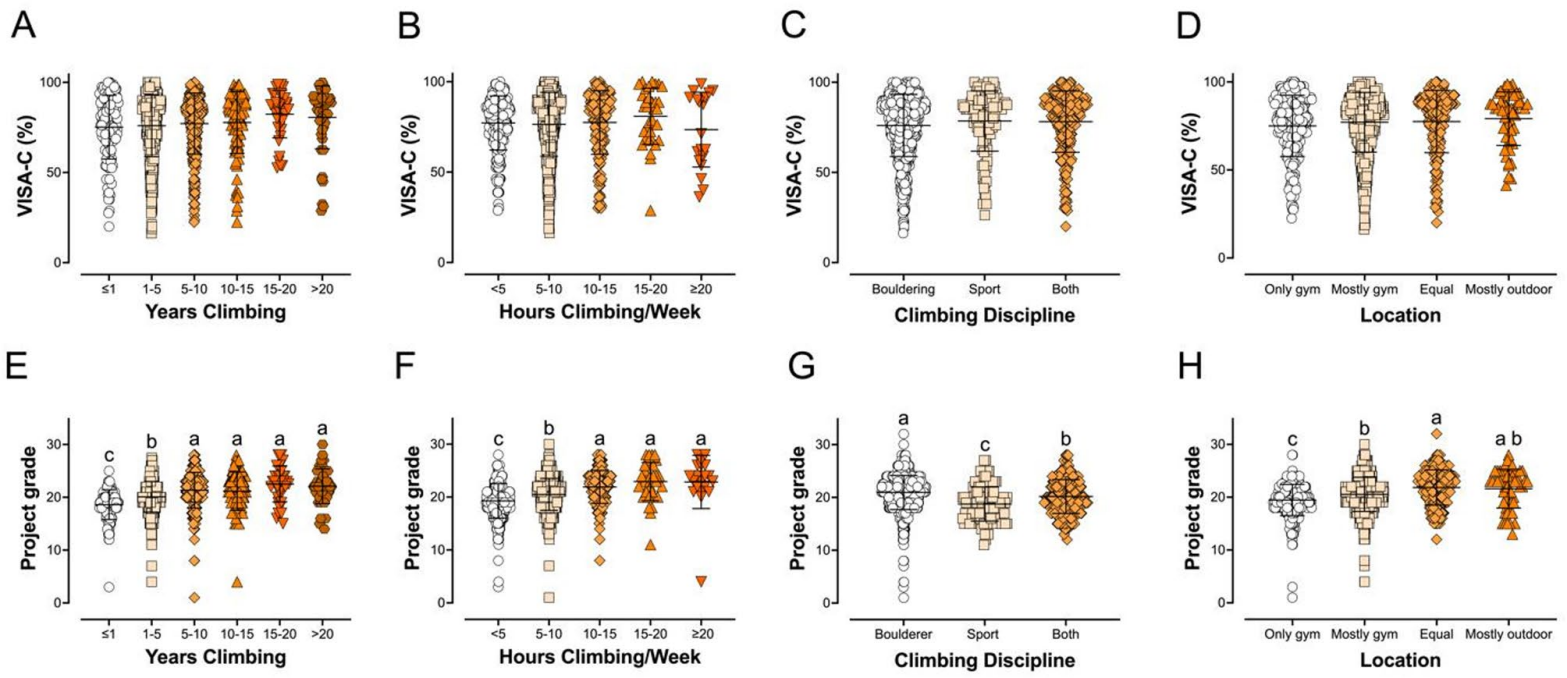
Sport-specific differences in VISA-C score and skill level. Years of climbing experience (**A** and **E**), the average number of hours per week over the last 6 months (**B**, **F**), the preferred climbing discipline (primarily bouldering, primarily sport/top-rope, or both roughly equally; **C**, **G**), and primary location (only in the gym, mostly in the gym, equal gym and outdoors, or mostly indoor; **D**, **H**) were compared. (**A**) VISA-C score improved with years of climbing experience (correlation *p* = 0.0045; one-way ANOVA with the groups in the figure trended toward significance with *p* = 0.0851; none of the groups were statistically significant with Tukey’s multiple comparisons test). (**B**) Hours spent climbing per week was not correlated with VISA-C score (correlation *p* = 0.6067 and one-way ANOVA *p* = 0.4870). Neither preferred (**C**) climbing discipline nor (**D**) location showed differences in VISA-C score (one-way ANOVA *p* = 0.1239 for discipline and *p* = 0.2129). (**E**) Years of climbing experience was positively correlated with project grade (correlation *p* < 0.0001; The one-way ANOVA with the groups in the figure was also significant, with *p* < 0.0001). (**F**) There were differences in project grade for the hours climbing per week using the bins in the figure (one-way ANOVA *p* < 0.0001). (**G**) Boulderers had the highest, those who did both had the second highest, and sport climbers had the lowest project grades; all three groups were statistically different (one-way ANOVA *p* < 0.0001) and with significant pairwise differences between all three groups. (**H**) Respondents who prefer to climb outdoors vs. indoors to varying degrees had different skill levels, with the indoor climbers being less skilled on average than those who did some or most of their climbing outdoors (one-way ANOVA *p* < 0.0001)

There was a positive association between years climbing and VISA-C score (*p =* 0.0045) but when the participants were broken down into bins (≤ 1 year, 1–5, 5–10, 10–15, 15–20, and > 20 years of experience; Fig. 5A) there were no differences between groups. Neither simple correlation (*p* = 0.6067) nor analysis between groups (*p* = 0.4870; Fig. 5B) yielded significant relationships between VISA-C score and hours spent climbing per week. Bouldering (*n* = 754) was the most popular discipline within our respondents, with fewer primarily lead climbing or top-roping (*n* = 114) and some doing both roughly equally (*n* = 241). Climbing discipline was not related to VISA-C scores (*p* = 0.1239). We also asked where our respondents typically climb. They responded with one of four answer options: only in the gym (*n* = 280), mostly gym climbing (*n* = 585), equal gym and outdoor climbing (*n* = 191), or mostly outdoors (*n* = 53). VISA-C scores were not different between these groups (*p* = 0.2129; Fig. 5D).

Years of experience climbing was a significant predictor of skill level (*p* < 0.0001; Fig. 5E), with skill level progressively increasing in each group up until 5–10 years of experience and no difference between groups past that. There was an increase in project grade with time spent climbing overall (*p* < 0.0001), with skill level increasing significantly among groups up until 10–15 h per week and then leveling off (Fig. 5F). Climbers of all three disciplines had distinct mean project grades (*p* < 0.0001). Boulderers had the highest average project grade, those who mostly did sport and top rope had the lowest, and climbers who did both equally were in the middle (Fig. 5G). Location was a predictor of project grade (*p* < 0.0001), with the stronger climbers spending some time training outdoors (Fig. 5H).

## Discussion

In this study, we tested a self-administered 8-question poll (which we named the VISA-C; Fig. 1) targeted to assess finger, hand, and wrist pain in rock climbers, based on the severity of pain and the limits on climbing intensity and volume caused by the pain. Climbers who reported no pain scored higher, on average, than those who reported pain, and climbers who reported pain which caused them to modify loading was the group with the lowest scores. As hypothesized , we saw stepwise decreases in VISA-C scores in the Pain and Limiting Pain groups. Observing good separation in VISA-C scores between these three groups indicates this survey was successful in its primary intended function. We also wanted to know whether VISA-C could be compared across climber demographics, so we asked participants to fill out additional demographic, health, lifestyle, and climbing history questions. VISA-C scores were not statistically different among any of the groupings we compared (age, sex, climbing ability, etc.,) except for blood pressure, for which the relationship observed was higher VISA-C scores (less pain/fewer tendon injuries) in individuals with elevated vs. normal BP.

All data collection in this study was anonymous. We recruited a diverse set of participants from six continents (Table 1). In some ways our sample is ideal: we had data from 1,110 total participants from 54 different countries. In others, our study population was far less than ideal. The most important of these were that 90% of respondents were men and only 10% were women, and we had only 3 climbers who identified as Black. The imbalance between men and women is seen throughout the literature. While men and women climb at roughly equal rates, men may perform more resistance training and have different motivations and training goals [7]. We also suspect that men are more likely to watch YouTube videos about climbing training and since this was the primary form of recruitment, we fell far short of our goal of equal recruitment. Our most popular climbing discipline was bouldering, which may have also been influenced by the mode of recruitment. However, boulderers may be more likely to perform supplemental finger training (this was observed in our retrospective study of training data from climbers using the Crimpd app [8]) and perhaps be more attracted to online climbing training content. The lack of racial diversity likely reflects a real lack of diversity in the sport.

### VISA-C Questionnaire Testing

With regard to the primary outcome of the study, we were able to detect statistically significant differences in VISA-C scores of each of the three pain groups (No Pain, Pain, and Limiting Pain; Fig. 3) with group averages of 83.2% (No Pain), 72.3% (Pain) and 60.1% (Limiting Pain). Though we observed significant group separation, the spread of the data within each group was large, with overlap between groups. We believe this is potentially due to factors including individual differences in pain perception and variation in how individuals interpret the questions. We do believe the VISA-C is a useful tool to gauge current pain levels. However, this questionnaire should not be used alone to diagnose injury without further independent and injury-specific validation.

As there were no significant differences between project grade and VISA-C score (Fig. S1A), we believe this survey can be administered reliably across all climbing ability levels. VISA-C scores were not significantly different in climbers who had done either no finger-specific training, low-intensity training, or high/maximal intensity finger training, either within the whole study population or in climbers with ≤ 1 year of climbing experience. These results are evidence that supplemental finger training may not be inherently riskier than climbing alone (Fig. S1B), even for beginner climbers (Fig. S1C).

Connective tissue adaptations occur in the fingers of climbers, but these adaptations are suggested to occur slowly, over a scale of years [9, 10]. For example, Schreiber and colleagues [10] demonstrated measurable increases in the thickness of A2 and A4 annular pulleys, the flexor digitorum superficialis (FDS), and flexor digitorum profundus (FDP) tendons, and the thickness of the proximal interphalangeal (PIP) and distal interphalangeal (DIP) joints, in climbers with 15 years or more of experience compared to non-climbers. We separately analyzed VISA-C scores in climbers with ≤ 1 year of climbing to address the ongoing discussion on whether targeted finger training is safe for newer climbers who have not had sufficient loading histories to develop detectable adaptations to the connective tissue of the finger flexor system. In this subset of climbers (*n* = 125), we did not see noticeable differences in VISA-C scores as a function of supplemental finger training. Our retrospective study [8] and several other studies [11, 12] have demonstrated that off-the-wall training can lead to more rapid strength gains compared to climbing alone, and may also accelerate physiologic adaptation [13] and prevent injury [14]. This principle aligns with the Training-Injury Prevention Paradox [15] which describes how athletes exposed to higher loads during regular training (with an optimal intermediate or “sweet spot” of acute: chronic workload ratio) are protected against injury: i.e., undertraining may increase injury risk just as much as overtraining. A climbing-specific analogy of this principle is when a climber encounters a strenuous move that exceeds their normal pulling intensity. If that individual does not regularly train their fingers at high loads, and the move exceeds the capacity of the tendons and pulleys, this can result in tissue strain or injury. Since we find no evidence of increased pain scores in beginner climbers who have a history of performing off-the-wall finger training, our analysis indicates (against common wisdom) it may be safe for beginners to perform supplemental training, particularly if done cautiously and avoiding overloading the relatively untrained musculoskeletal tissues of the upper body. Various finger training regimens geared toward more advanced climbers exist, but these programs may need to be modified to allow progressive increases in off-the wall loading to help novice climbers improve their climbing ability and potentially reduce injury risk.

As data on injury risk factors is limited and often conflicting, as detailed in a systematic review [16] by Quarmby, et al., we sought to determine whether we could detect any correlations between current pain levels and the other demographic data we collected and extrapolate relative injury risk from those associations. Incorporating data collected from our respondents in our supplemental questionnaire, we next used VISA-C scores to make comparisons of finger health between different demographic groups, health parameters, and training history. While the VISA-C is only a measure of current finger pain for an individual and does not reflect the injury history of the climber, we used it in the present study to search for potential physiological insight that may come from using correlation and multiple-group statistical analysis.

### Respondent Demographics

In our subject pool, men performed on average 1.7 IRCRA project grades higher than women (Fig. 2D). Regardless of the grade, hand and wrist pain levels were not different as a function of biological sex (Fig. 2A). A review by Quarmby, et al. [16] notes conflicting data regarding sex as an injury factor. One such study found that while females had a higher incidence of shoulder/upper arm injuries than males, there was no sex differences in the incidence of other upper extremity injuries [17].

Across our participants, who ranged from 18 to 50 years of age, VISA-C scores were not significantly different as a function of age. We did see small but statistically significant decreases in climbing ability with age. The group with the highest average project grade was the 20–25–year-olds (21.3) and the lowest was in the 31–35–year-olds (19.7), but most groups did not reach significance upon pairwise comparison. We believe that these data indicate there is a small decrease in ability with age, but these results are confounded by the positive effects of training and increasing number of years of experience in the sport.

Climbing skill was not different between races (Fig. 2F). Although there were trends for VISA-C score to differ as a function of race, our data did not reach significance (Fig. 2C). Tendon injury rates can differ as a function of race [18]. However, the difference identified in previous work was that Black individuals were significantly more likely than Whites to suffer Achilles, quadriceps, or patellar tendon ruptures. The small sample of black climbers who participated, made this comparison impossible in our study. The strongest tendency in the current work was for Hispanic and white men to have lower VISA-C scores than the other groups. However, the differences were small and non-significant.

### Health and Lifestyle

Though BMI is an inaccurate measure of health and fitness since it does not consider body composition or lean mass percentage, we use it here to represent the size of the climber. Neither height nor weight individually (data not shown), nor the two variables combined as BMI, were associated with significant differences in VISA-C score (Fig. 2C). There is an ongoing debate over what degree height (and arm reach) and weight contribute to climbing ability. Our results indicate that for forearm/finger injury risk, body weight is of limited concern.

Climbing, however, is a strength-to-weight ratio sport, with elite climbers being leaner than elite athletes of other sports. If fact, elite climbers may be at higher risk for relative energy deficiency in sport (RED-S), and eating disorders [19]. The high prevalence of RED-S in climbing is often driven by a desire to optimize the strength-to-weight ratio and maximize performance. A review on the determinants of climbing success by Saul, et al. [20] found negative correlations between weight and climbing ability as measured by red-point level, and that elite climbers had a lower body fat percentage than recreational climbers, but that in their study a lower BMI was not significantly associated with higher climbing level across the studies they analyzed data from. The mean BMI of climbers in our study was 22.7, which is in the middle of the range considered “healthy”, much leaner than the average adult population. Our results show that for our participants, BMI did not negatively affect the project grade within the “healthy” weight range; only “overweight” groups showed significant differences in skill using pairwise comparisons (Fig. 4D).

The trend toward significance on VISA-C score with diet (*p =* 0.0686 comparing meat eaters, vegetarians, and vegans; Fig. 4B) may indicate that diet can influence injury risk to small degree, but whether this is due to eating meat and animal products, or a higher likelihood of vegetarians and vegans to be more focused on health more generally is unknown. The difference between vegetarian/vegan climbers and omnivores is likely the result of vegetarians and vegans living healthier in general, as they are more likely to engage in leisure-time physical activity and get enough sleep [21]. Maybe somewhat surprisingly, diet did not scale with project grade (Fig. 4E).

Perhaps the most unexpected observation was the relationship between blood pressure (BP) and VISA-C score: we observed higher VISA-C scores (less pain and injury) in individuals with elevated BP (Fig. 4C). We were not able to distinguish an effect of high BP against either normal or elevated BP because there were very few respondents in the high BP group (*n* = 12). Unlike VISA-C, we did not observe an association between BP and project grade, suggesting that this relationship between BP and VISA-C was not a function of selection bias (Fig. 4F). We included questions on BP in the study since there is growing interest in the role of angiotensin II (Ang II) in tendon function. Ang II is a hormone belonging to the renin-angiotensin-aldosterone system (RAAS) that controls blood pressure, fluid balance, and electrolyte levels in the body through regulation of vascular tone [22]. Ang II exerts its effects in part by activating the Ang II type 1 receptors (AT_1_Rs), which belong to G-protein coupled receptor (GPCR) family. In addition to its effects on blood pressure, AT_1_R activation induces cell-survival responses including activation of transforming growth factor-β (TGF-β) and increased collagen secretion [23]. In pathological states, chronic excess Ang II activity can contribute to tissue fibrosis, but little is known on the effects of Ang II on connective tissues, which are comprised primarily of collagen matrices. In contrast to elevated Ang II, drugs that block the AT_1_R are associated with a 7.6-fold increase in tendon rupture [24] suggesting that AT_1_R activity is important for tendon function. In support of a positive role of Ang II, one of the best genetic predictors of elite performance in strength and power athletes is the presence of the D allele of the angiotensin converting enzyme [25]. Individuals with the ACE D polymorphism have slightly elevated Ang II levels and the effect on tendon collagen and stiffness may underlie the positive effect of this hormone on performance and connective tissue health observed in this study. Therefore, the tendency for climbers in our study with elevated blood pressure to have higher VISA-C scores may have a physiological basis that involves Ang II/AT_1_Rs.

### Climbing History and Habits

Training is the most important determinant of climbing performance. A study by Mermier and colleagues from 2000 [26] that aimed to identify the physiologic and anthropometric determinants of climbing performance ran a principal component analysis (PCA) to detect variables associated with variance and then performed regressions to determine that trainable variables (those that are influenced by training such as strength, power, and percentage body fat) were associated with the highest amount (58.9%) of variance in climbing performance, versus 0.3% for anthropometric variables and 1.8% for flexibility. Therefore, at least as explained by this model, climbing performance is largely trainable and modifiable rather than predetermined by a particular set of anthropometric characteristics, consistent with the fact that in our study, climber anthropometry had little relation with either VISA-C or project grade.

Years of climbing experience was positively correlated with VISA-C score, but the increase was minimal and when we split the respondents into experience brackets (≤ 1 years, 1–5 years, then 5-year increments), we did not find meaningful differences between groups (Fig. 5A). Overall, there was a positive association with years of climbing with project grade. We observed progressive increases from ≤ 1 years to 1–5 years and to 5–10 years, but we did not see additional improvements with increasing experience beyond that. This is not to say that individuals may not keep improving beyond this timeframe, especially if they implement changes to their climbing intensity, frequency, or training methods, but on the population level we saw that project grade stabilizes around 5–10 years of experience. This association reflects the increases in sport-specific ability gained from training, which simultaneously leads to adaptations in both strength gains and improvements in skill or coordination.

Most climbers spent between 5 and 10 h climbing per week (*n* = 635). The number of hours participants spent climbing did not significantly impact VISA-C score, either as a correlation or between binned groups (Fig. 5B). Climbing 20 + hours per week was associated with the lowest (73.5%) VISA-C score, indicating potential negative effects of overtraining, but there were the fewest participants in this group (*n* = 20), making it difficult to draw statistical conclusions.

While VISA-C scores were not significant across preferred climbing discipline (bouldering vs. sport/top-rope vs. both equally; Fig. 5C) there were significant strength differences between groups (Fig. 5G). Specifically, boulderers had the highest IRCRA project grades. This agrees with the literature where boulderers have been shown to be superior in all metrics of strength and power [27]. Compared with similarly graded sport routes, which are more endurance-based, bouldering problems generally require higher intensity and strength. Athletes may self-select the disciplines that best fit their body types, with strength or power athletes perhaps preferring bouldering, and endurance-type athletes leaning toward lead climbing.

Whether a climber climbs in the gym or outside had no impact on VISA-C score (Fig. 5D) but did impact climbing skill level (Fig. 5H). In our study, those who climbed outdoors at least some of the time had higher project grades. This is another variable that is difficult to interpret but may be influenced by the higher accessibility of gyms for beginner climbers, with climbers with more experience and/or higher skill levels venturing outdoors more often. It is possible that varying setting and climbing both in the gym and outdoors contributes to improved coordination and climbing skill.

## Limitations

The limitations of this study lie primarily in the self-administered survey study design we used and the potential for participant recruitment bias. As with all surveys, we cannot verify the accuracy of information provided by the respondents. It is possible that respondents did not properly understand some of the questions we asked. Ideally, we would have had clinical confirmation of a diagnosis as part of the study; however, given the large cohort and their global distribution this was not possible. Future work should incorporate a comparison of VISA-C scoring and in person confirmation of diagnosis and should also compare VISA-C scores over time as a function of normal activity versus interventions designed to decrease pain. In this way, we would be able to better understand test: retest reliability and the individual consistency of the measure. The primary bias was that we asked secondary health/anthropometry questions based on processes or hypotheses that we had *a priori*. This means the questions were biased by the investigators beliefs as to which physiological factors may influence injury rate. The second bias was that we recruited climbers who were using supplemental training or watching YouTube videos on supplemental training practices. We strongly suspect that a majority of the participants were brought to our survey by a recent YouTube video on Emil Abrahamsson’s channel on retrospective hangboard training [8] in which the survey for the present study was mentioned and a link to the study provided; however, this strategy allowed us to obtain a large subject data set. Also, as the survey was only available in English, our exclusion of non-English speakers potentially biased our sample. Lastly, we relied on simple correlations and comparisons between groups for our secondary analyses, and it is unknown whether there were any underlying and unidentified confounding variables that affected our analyses. We also failed to address whether participants performed other sports that might result in finger/wrist injuries.

## Applications

Potential applications for the VISA-C assessment are broad, ranging from assessing whether an individual is healthy enough, for example, to responsibly participate in maximal load training, or tracking finger health over time as an individual recovers from an injury or during a training cycle to ensure the athlete is not compromising tissue health through overexertion. Separate longitudinal studies are needed to further validate the survey against the progression of climbing-related injuries to specific tissues, which may have unique recovery timelines. Different injuries may also have unique limits on performance (i.e., limits on loading only specific grip types or anatomical positions).

## Conclusions

We created and tested the ability of a novel self-administered 8-question questionnaire, the VISA-C, to quantify pain levels of the fingers, hands, and wrists in climbers and the limits this pain had on climbing training intensity and duration. This study looked at data from 1,110 climbers. VISA-C score scaled with pain, suggesting that the questionnaire was effective at identifying individuals who may do better with modified training. We analyzed pain scores and climbing skill level across this population to make correlations with various factors, from data gathered from each participant in a supplemental questionnaire, that are traditionally associated with climbing performance. We believe it is appropriate to use the VISA-C broadly, since scores were consistent across all major demographics. One of the most surprising takeaways from the secondary analyses from the supplemental questionnaire, was that even though several of the variables correlated with VISA-C score (our proxy for current finger pain levels) or an individual’s IRCRA project grade, none were associated with both VISA-C and project grade. Also, some of the associations we did observe in our data were surprising with interesting physiologic implications, such as the higher VISA-C scores in individuals with elevated BP, pointing to interesting physiological implications that warrant further investigation.

## Supplementary Information

Below is the link to the electronic supplementary material.

Supplementary Material 1

## Data Availability

Raw data are available on request from the corresponding author.

## Abbreviations

Ang II: Angiotensin II
AT_1_Rs: Ang II type 1 receptors
BMI: Body mass index
BP: Blood pressure
CLD: Compact letter display
RAAS: Renin–angiotensin–aldosterone system
RED-S: Relative energy deficiency in sport
VISA: Victorian Institute of Sports Assessment
VISA-A: Victorian Institute of Sports Assessment Scale for Achilles Tendinopathy
VISA-P: VISA Scale for Patellar Tendinopathy
VISA-C: Victorian Institute of Sports–like Assessment; C for climbing
IRCRA: International Rock–Climbing Research Association
