# Supplementary material for: The VISA-C Questionnaire: A Self-Administered Assessment to Measure Finger/Hand/Wrist Pain in Climbers
Source: Sports Med Open. 2025 Oct 1;11:105. doi: 10.1186/s40798-025-00912-y (PMC12488532; doi:10.1186/s40798-025-00912-y)
Supplement: Supplementary file 1 — Supplementary Material 1 [file 40798_2025_912_MOESM1_ESM.docx]

Sports Medicine – Open

**Validation of the VISA-C Questionnaire: a Self-Administered Assessment to Measure Finger/Hand/Wrist Pain in Climbers**

Natalie K. Gilmore^1^, Peter Klimek^2^, Emil Abrahamsson^3^, and Keith Baar^1,4,5^

^1^Department of Neurobiology, Physiology and Behavior, University of California Davis, Davis, CA 95616

^2^Crimpd, Inc. Seattle, WA 98118, USA

^3^Ängarna 347, 837 98, Sweden

^4^Department of Physiology and Membrane Biology, University of California Davis, Davis, CA 95616

^5^VA Northern California Health Care System, Mather, CA 95655, USA.


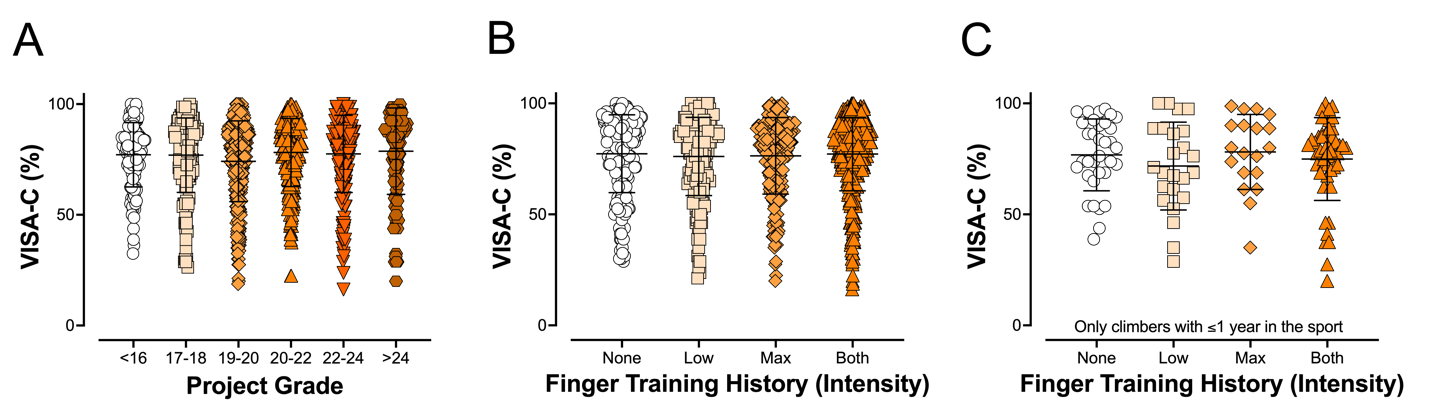


**Figure S1.** VISA-C score is not different with (A) climbing skill level (project grade using the IRCRA scale) or (B and C) recent (past 6 month) history of finger training. (A). We performed both correlation of project grade vs. VISA-C (*p =* 0.057) and one-way ANOVA using the bins in the figure (*p =* 0.3388). Respondents were asked if they have performed either high intensity (max), low intensity (low), or both types of finger-specific training in the past 6 months. These groups were compared for (B) all respondents (*p* = 0.8292) as well as for (C) climbers with ≤ 1 year of climbing experience (*p* = 0.6510).
